# Influence of pelleting and β-mannanase on nutrient digestibility and metabolizable energy of DDGS, copra meal, palm kernel meal, and jatropha meal in precision-fed Single Comb White Leghorn roosters

**DOI:** 10.1371/journal.pone.0355743

**Published:** 2026-08-07

**Authors:** Bounmy Keohavong, Musabbir Ahammed, Jong Suh Shin, Lohakare Jayant, Sang Jip Ohh

**Affiliations:** 1 Department of Animal Science, Souphanouvong University, Luang Prabang, Laos; 2 Department of Poultry Science, Bangladesh Agricultural University, Mymensingh, Bangladesh; 3 Department of Animal Science, Kangwon National University, Chuncheon, Republic of Korea; 4 Department of Animal Science (Agriculture), University of Arkansas at Pine Bluff: Pine Bluff, Arkansas, United States of America; 5 Department of Animal Science, Kangwon National University, Chuncheon-si, Republic of Korea; Universitas Sebelas Maret, INDONESIA

## Abstract

This study evaluated the effects of pelleting and β-mannanase supplementation on nutrient digestibility and metabolizable energy of four alternative feed ingredients distillers dried grains with solubles (DDGS), copra meal, palm kernel meal (PKM), and jatropha meal (JM) using precision-fed Single Comb White Leghorn roosters. Two independent precision-fed rooster assays were conducted. In Experiment 1, each ingredient was offered in mash form or as pellets produced under controlled conditions (<65 °C) to minimize heat-induced nutrient damage. In Experiment 2, mash samples were supplemented with β-mannanase (0.25% as-fed basis). Apparent dry matter digestibility, nitrogen retention, and metabolizable energy values, including apparent metabolizable energy (AME), nitrogen-corrected AME (AMEn), true metabolizable energy (TME), and nitrogen-corrected TME (TMEn), were determined. Pelleting improved dry matter digestibility, nitrogen retention, and all metabolizable energy parameters across ingredients (p < 0.05), with more pronounced responses observed in DDGS and jatropha meal. β-mannanase supplementation also enhanced nutrient utilization and energy utilization, particularly in copra meal and palm kernel meal, which are rich in β-mannan-containing non-starch polysaccharides. Overall, the results demonstrate that both pelleting and β-mannanase supplementation can improve the nutritional value of fibrous alternative feed ingredients, thereby supporting their more efficient use in poultry diets.

## Introduction

The increasing global demand for poultry products has intensified pressure on conventional feed ingredients such as corn and soybean meal, which are associated with high costs, supply instability, and environmental concerns [1]. These challenges are particularly pronounced in tropical and developing regions, where feed resources are often limited and production systems must rely on locally available alternatives. Consequently, there is growing interest in the utilization of agro-industrial byproducts as sustainable and cost-effective feed ingredients for poultry production. Among these, distillers dried grains with solubles (DDGS), copra meal (CM), palm kernel meal (PKM), and jatropha meal (JM) have attracted considerable attention due to their wide availability and relatively low cost.

Distillers dried grains with solubles (DDGS), a coproduct of the bioethanol industry, is widely incorporated into poultry diets as a source of energy and protein. However, its nutritional value is often inconsistent due to variability in raw materials and processing conditions, and its relatively high fiber content can limit nutrient digestibility and metabolizable energy utilization in poultry [2,3]. Similarly, oilseed byproducts such as copra meal and palm kernel meal are abundant in tropical regions and represent economically viable feed resources. Despite these advantages, their utilization in poultry diets is constrained by high concentrations of dietary fiber and non-starch polysaccharides (NSPs), particularly β-mannans, which reduce nutrient digestibility and metabolizable energy availability [4]. These NSPs increase intestinal viscosity and impair nutrient absorption, thereby limiting feed efficiency and overall performance [5].

Jatropha meal, derived from *Jatropha curcas*, is another promising alternative protein source due to its high crude protein content and potential availability in tropical regions. However, its application in poultry nutrition remains limited because of the presence of antinutritional and toxic compounds, particularly phorbol esters, which adversely affect animal health and performance unless adequately detoxified [6]. In addition, information regarding its metabolizable energy and nutrient digestibility in poultry is still scarce, particularly under different processing conditions, highlighting the need for further systematic evaluation.

Dietary fiber in alternative feed ingredients presents both nutritional challenges and functional opportunities in poultry feeding. While excessive fiber generally reduces nutrient utilization, moderate inclusion levels may promote gut health, microbial balance, and intestinal development [5,7]. Nevertheless, poultry lack endogenous enzymes capable of efficiently degrading NSPs, which limits the effective utilization of fibrous feed ingredients. Therefore, strategies that enhance fiber degradation and nutrient availability are essential for improving the feeding value of these alternative resources.

Feed processing technologies and exogenous enzyme supplementation have been widely explored as practical approaches to overcome these limitations. Pelleting, one of the most common feed processing methods, can improve feed handling characteristics, reduce ingredient segregation, and enhance nutrient digestibility through physical and thermal modification of feed components. In parallel, exogenous enzymes such as β-mannanase have been shown to hydrolyze β-mannans into simpler carbohydrates, thereby reducing digesta viscosity and improving nutrient absorption and energy utilization. Recent studies have demonstrated that β-mannanase supplementation enhances growth performance, nutrient digestibility, and metabolizable energy in poultry, particularly when diets are rich in fibrous ingredients [3,8]. However, the extent of these improvements depends on multiple factors, including ingredient type, processing conditions, and enzyme inclusion levels.

Despite considerable research on individual feed ingredients and nutritional interventions, comparative evaluations of multiple alternative feed ingredients under standardized experimental conditions remain limited. In particular, there is a lack of comprehensive data on how pelleting and β-mannanase supplementation influence both nutrient digestibility and metabolizable energy, including apparent and true values, across different fibrous feed ingredients when assessed using precision-fed rooster assays. Such information is critical for improving feed formulation accuracy and optimizing the utilization of alternative feed resources in poultry production systems.

Therefore, the present study was conducted to evaluate the influence of pelleting and β-mannanase supplementation on nutrient digestibility and metabolizable energy of DDGS, copra meal, palm kernel meal, and jatropha meal in poultry. It was hypothesized that pelleting and β-mannanase supplementation would enhance nutrient utilization and energy availability, with responses varying according to the physicochemical characteristics of each ingredient.

## Materials and methods

### Ethical approval

The experimental protocol was reviewed and approved by the Institutional Animal Care and Use Committee of Kangwon National University, Chuncheon, Republic of Korea (Approval No. KW-210707–1). All experimental procedures involving animals were conducted in accordance with the institutional guidelines for the care and use of laboratory animals and complied with internationally accepted principles for animal research ethics. There were no deviations from the approved study protocol after ethical approval was obtained.

### Experimental ingredients and sample preparation

Four alternative feed ingredients were evaluated: distillers dried grains with solubles (DDGS), copra meal (CM), palm kernel meal (PKM), and jatropha meal (JM). Samples of DDGS, CM, and PKM were obtained from Hanilfeed Co. (Korea), whereas JM was sourced from KOLAO Co. (Lao People’s Democratic Republic). The chemical composition and pellet hardness of the experimental ingredients are presented in Table 1.

**Table 1:** Nutrient composition and pellet hardness of distillers dried grains with solubles (DDGS), copra meal (CM), palm kernel meal (PKM), and jatropha meal (JM).

Variable	DDGS	CM	PKM	JM
Dry matter (g/kg)	849.80	922.29	923.70	922.70
Gross energy (MJ/kg)	19.54	21.08	22.41	24.22
Nitrogen (g/kg)	35.44	30.42	22.57	27.42
Crude protein (g/kg)	221.52	190.12	141.04	171.41
Ether extract (g/kg)	49.24	29.56	66.50	79.65
Ash (g/kg)	68.17	62.58	48.66	48.48
Organic matter (g/kg)	781.64	859.71	875.04	874.21
Total carbohydrate (g/kg)	510.88	640.03	667.50	623.15
Pellet hardness (N)	24.59	87.31	105.92	42.05

*Note:* Pellet hardness measured in Newton (N). All values are expressed on an as-dry matter basis.

For Experiment 1, all ingredients were ground to pass through a 1-mm screen and prepared in either mash or pelleted form. Pelleting was performed using a pellet mill (Model CPP020, Chia Tung Development Corp., Taiwan; capacity 275.55 kg/h) equipped with a die measuring 4 mm in diameter and 45 mm in thickness. The resulting pellets had an approximate diameter of 4 mm and were cut to a length of approximately 5–10 mm. The conditioning temperature was maintained below 65 °C to minimize heat-induced nutrient degradation.

Pellet hardness was determined for pelleted samples using a digital force gauge (Model HLD, Handpi Instruments, China) following Svihus et al. [9]. Ten pellets per ingredient were randomly selected, and the maximum force required to fracture each pellet was recorded and expressed in Newtons (N).

For Experiment 2, a commercial β-mannanase enzyme was incorporated into the mash form of each ingredient at 0.25% (as-fed basis), whereas unsupplemented mash samples served as controls. All prepared samples were stored in sealed containers at ambient temperature until use.

### Birds, housing, and experimental design

Twenty non-cecectomized Single Comb White Leghorn roosters (Gallus gallus domesticus), 32 weeks of age with a mean body weight of 2,602 ± 85 g, were used. Birds were individually housed in stainless-steel metabolic cages (50 × 40 × 50 cm) equipped with feeders and nipple drinkers and maintained in a temperature-controlled room (25.2 ± 1.3 °C). Birds were acclimated for 10 days with ad libitum access to water and a commercial diet.

Non-cecectomized roosters were used because the precision-fed rooster assay described by Sibbald [10] has been extensively validated for determining metabolizable energy values of feed ingredients in poultry. The use of intact birds maintains normal gastrointestinal physiology and avoids potential alterations associated with surgical cecectomy. Furthermore, the primary objective of the present study was to compare the effects of feed ingredient type, physical processing, and β-mannanase supplementation on nutrient utilization and metabolizable energy rather than to determine amino acid digestibility. Therefore, the use of non-cecectomized roosters was considered appropriate and consistent with established methodologies for ingredient evaluation in poultry nutrition research.

Two independent experiments were conducted:

- Experiment 1: 4  ×  2 factorial arrangement consisting of four feed ingredients (DDGS, CM, PKM, and JM) and two physical forms (mash and pellet).
- **Experiment 2:** 4 × 2 factorial arrangement consisting of four feed ingredients (DDGS, CM, PKM, and JM) and two β-mannanase supplementation levels (0 and 0.25%).

### Precision-fed metabolizable energy assay

Apparent metabolizable energy (AME), nitrogen-corrected apparent metabolizable energy (AMEn), true metabolizable energy (TME), and nitrogen-corrected true metabolizable energy (TMEn) were determined using the precision-fed rooster assay described by Sibbald [10], with modifications outlined by Dudley-Cash [11].

Prior to each assay, roosters were fasted for 24 h to ensure complete evacuation of gastrointestinal contents. Test ingredients were individually administered by crop intubation at a fixed amount of 30 g (as-fed basis) per bird. Ingredients were fed as single test materials without dilution or mixing with a reference diet.

Each experiment was arranged as a 4 × 2 factorial design comprising eight treatment combinations. The assay was conducted over seven consecutive experimental rounds, with each round considered an experimental block. During each round, 16 roosters were allocated to the eight treatment combinations, providing two birds per treatment. An additional four roosters were maintained in a fasted state throughout the collection period to estimate endogenous energy and nitrogen losses required for the calculation of true metabolizable energy values. Thus, a total of 20 roosters were used in each experimental round.

Following crop intubation, birds had free access to water but no feed during the 48-h collection period. Total excreta were quantitatively collected from each bird for 48 h, immediately frozen at −80°C, and subsequently freeze-dried prior to chemical analyses. Endogenous energy and nitrogen losses were determined from excreta collected from the fasted birds and were used to calculate true metabolizable energy values.

The same roosters were reused across experimental rounds following an adequate recovery period during which birds were returned to ad libitum feeding and normal husbandry conditions. Treatment assignments were rotated among rounds to minimize potential bird and period effects. Experimental round was included as a random blocking factor in the statistical analysis to account for variation among assay periods.

### Chemical composition and laboratory analyses

Feed ingredients and freeze-dried excreta samples were analyzed in triplicate according to standard methods of AOAC International [12]. Dry matter (DM) was determined using AOAC Method 930.15, crude ash using AOAC Method 942.05, ether extract (EE) using AOAC Method 920.39A, and crude protein (CP) using AOAC Method 988.03. Nitrogen content was determined by the Kjeldahl method, and crude protein was calculated as nitrogen × 6.25.

Gross energy (GE) was determined using an adiabatic bomb calorimeter (Model 1261, Parr Instrument Company, Moline, IL, USA). Organic matter (OM) was calculated as the difference between dry matter and ash content [12], whereas total carbohydrate (CHO) content was estimated by difference according to the following equation:


$$\begin{array}{c} CHO=DM-(CP+EE+Ash) \end{array}$$ (1)

Nutrient intake and nutrient output were calculated from the nutrient concentrations of the test ingredients and excreta. Dry matter digestibility (DMD), apparent nitrogen retention (ANR), true nitrogen retention (TNR), and apparent total tract retention coefficients of DM, GE, nitrogen, CP, EE, ash, OM, and CHO were subsequently determined.

Apparent nitrogen retention was calculated as the difference between nitrogen intake and nitrogen excretion, whereas true nitrogen retention was corrected for endogenous nitrogen losses measured in fasted birds. Apparent metabolizable energy (AME) and nitrogen-corrected apparent metabolizable energy (AMEn) were calculated from gross energy intake and excreted energy. True metabolizable energy (TME) and nitrogen-corrected true metabolizable energy (TMEn) were corrected for endogenous energy and nitrogen losses determined from the fasted control birds. All metabolizable energy calculations were performed according to the procedures described by Sibbald [10].

## Statistical analysis

Data from Experiments 1 and 2 were analyzed separately using the MIXED procedure of SAS software (Version 9.2; SAS Institute Inc., Cary, NC, USA). For each experiment, data were analyzed using the following mixed model:


$$\begin{array}{c} Yijk=\mu +Fi+Tj+(F\times T)ij+Bk+\epsilon ijk \end{array}$$ (2)

where Yijk is the observed response variable, μ is the overall mean, Fi is the fixed effect of feed ingredient, Tj is the fixed effect of treatment (physical form in Experiment 1 or β-mannanase supplementation in Experiment 2), (F × T)ij is the interaction between feed ingredient and treatment, Bk is the random effect of experimental round (block), and εijk is the residual error term.

Experimental round was included as a random blocking factor to account for variation among assay periods, and individual birds served as the experimental units. Least-squares means were compared using the PDIFF option of SAS when significant effects were detected. Results are presented as least-squares means with their standard errors of the mean (SEM), and statistical significance was declared at P < 0.05.

## Results

### Experiment 1: Effects of physical form on nutrient utilization and metabolizable energy

Tables 2–4 present the effects of feed ingredient, physical form (mash vs. pellet), and their interaction on nutrient intake, nutrient utilization, and metabolizable energy values. Overall, pelleting consistently improved nutrient availability and energy utilization; however, the magnitude of the response varied significantly among ingredients, as indicated by strong ingredient × physical form interactions across most variables.

**Table 2:** Experiment 1: Effects of physical form (mash vs pellet) on nutrient balance of distillers dried grains with solubles (DDGS), copra meal (CM), palm kernel meal (PKM), and jatropha meal (JM).

Variable	DDGS		CM		PKM		JM		Tested feedstuffs (TF)				Physical forms (PF)		SEM	*P*-value		
	Mash	Pellet	Mash	Pellet	Mash	Pellet	Mash	Pellet	DDGS	CM	PKM	JM	Mash	Pellet		PF	TF	PF × TF
Total nutrient intake (g)																		
Dry matter	25.18^h^	25.81^g^	27.31^e^	28.03^b^	26.90^f^	28.52^a^	27.56^d^	27.80^c^	25.49^b^	27.67^a^	27.71^a^	27.68^a^	26.74^b^	27.54^a^	0.52	<0.001	<0.001	<0.001
Gross energy, MJ	0.48^h^	0.52^g^	0.55^f^	0.62^d^	0.58^e^	0.67^b^	0.66^c^	0.68^a^	0.50^d^	0.58^c^	0.62^b^	0.67^a^	0.57^b^	0.62^a^	0.03	<0.001	<0.001	<0.001
Nitrogen	0.93^a^	.088^b^	0.83^d^	0.86^c^	0.57^h^	0.68^g^	0.78^e^	0.73^f^	0.90^a^	0.81^b^	0.63^d^	0.76^c^	0.78	0.79	0.05	0.437	<0.001	<0.001
Crude protein	5.78^a^	5.51^b^	5.16^d^	5.36^c^	3.60^h^	4.23^g^	4.89^e^	4.60^f^	5.65^a^	5.26^b^	3.92^d^	4.74^c^	4.86	4.92	0.30	0.411	<0.001	<0.001
Ether extract	0.80^g^	1.72^d^	0.58^h^	1.06^f^	1.62^e^	2.08^c^	2.24^a^	2.17^b^	1.26^c^	0.82^d^	1.85^b^	2.21^a^	1.31^b^	1.76^a^	0.31	<0.001	<0.001	<0.001
Crude ash	1.69^d^	1.79^b^	1.44^e^	2.03^a^	0.10^f^	1.72^c^	0.10^f^	1.69^d^	1.74^a^	1.73^a^	1.36^b^	1.35^b^	1.28^a^	1.81^a^	0.19	<0.001	<0.001	<0.001
Organic matter	19.45^g^	20.41^f^	23.42^d^	24.16^c^	23.14^e^	25.38^a^	24.32^b^	24.07^c^	19.93^c^	23.79^b^	24.26^a^	24.20^a^	22.58^b^	23.51^a^	0.89	<0.001	<0.001	<0.001
Total carbohydrate	12.87^f^	13.18 ^e^	17.67 ^c^	17.74 ^c^	17.93 ^b^	19.07^a^	17.19 ^d^	17.31^d^	13.03^d^	17.71^b^	18.50^a^	17.25^c^	16.42	16.83	0.87	0.106	<0.001	<0.001
Total nutrient output (g)																		
Dry matter	17.21^d^	15.98^e^	21.99^c^	22.76^bc^	24.12^a^	24.11^a^	23.29^ab^	22.29^bc^	16.59^c^	22.38^b^	24.12^a^	22.79^b^	21.65	21.29	2.53	0.147	<0.001	<0.001
Gross energy, MJ	0.30^e^	0.31^e^	0.42^d^	0.46^bc^	0.47^ab^	0.49^a^	0.44^cd^	0.43^d^	0.31^c^	0.44^b^	0.48^a^	0.44^b^	0.41^b^	0.42^a^	0.05	0.050	<0.001	<0.001
Nitrogen	1.14^c^	1.15^c^	1.12^c^	1.13^c^	1.17^c^	1.04^d^	1.68^a^	1.41^b^	1.14^b^	1.13^b^	1.11^b^	1.55^a^	1.28^a^	1.18^b^	0.16	<0.001	<0.001	<0.001
Crude protein	7.10^c^	7.18^c^	7.01^c^	7.07^c^	7.31^c^	6.49^d^	10.50^a^	8.84^b^	7.14^b^	7.04^b^	6.90^b^	9.67^a^	7.98^a^	7.39^b^	0.02	<0.001	<0.001	<0.001
Ether extract	0.15^g^	0.22^e^	0.47^d^	0.57^c^	0.77^b^	0.84^a^	0.06^h^	0.18^f^	0.19^c^	0.52^b^	0.80^a^	0.12^d^	0.36^b^	0.45^a^	0.13	<0.001	<0.001	<0.001
Crude ash	5.52^b^	3.53^f^	5.81^b^	4.73^d^	5.10^c^	4.10^e^	5.77^b^	8.53^a^	4.53^c^	5.27^b^	4.60^c^	7.15^a^	5.55^a^	5.22^b^	0.09	0.002	<0.001	<0.001
Organic matter	11.69^f^	12.44^f^	16.17^d^	18.04^c^	19.02^b^	20.03^a^	17.51^c^	13.77^e^	12.06^d^	17.11^b^	19.52^a^	15.64^c^	16.10	16.07	2.15	0.898	<0.001	<0.001
Total carbohydrate	4.43^g^	5.04^f^	8.69^d^	10.40^c^	10.93^b^	12.70^a^	6.96^e^	4.75^fg^	4.74^d^	9.54^b^	11.81^a^	5.85^c^	7.75^b^	8.22^a^	1.60	0.028	<0.001	<0.001
DMD (%), Apparent nitrogen retained (ANR) and True nitrogen retained (TNR)																		
DMD	31.67^b^	38.09^a^	19.48^c^	18.78^c^	10.35^d^	15.42^c^	15.50^c^	19.82^c^	34.88^a^	19.13^b^	12.88^c^	17.66^b^	19.25^b^	23.02^a^	8.73	<0.001	<0.001	<0.001
ANR	−0.21^a^	−0.27^ab^	−0.30^bc^	−0.27^ab^	−0.60^d^	−0.36^c^	−0.90^f^	−0.68^e^	−0.24^a^	−0.29^b^	−0.48^c^	−0.79^d^	−0.50^b^	−0.39^a^	0.17	<0.001	<0.001	<0.001
TNR	0.60^a^	0.54^ab^	0.51^bc^	0.54^ab^	0.22^d^	0.45^c^	−0.09^f^	0.13^e^	0.57^a^	0.53^b^	0.33^c^	0.02^d^	0.31^b^	0.42^a^	0.17	<0.001	<0.001	<0.001

*Note:* DMD = dry matter digestibility; ANR = apparent nitrogen retention; TNR = true nitrogen retention. Means with different superscripts within a row of each classification (physical form or physical form × ingredient interaction) differ significantly (P < 0.05). SEM = standard error of the mean.

**Table 3:** Experiment 1: Effects of physical form (mash vs pellet) on apparent total tract retention of nutrients in distillers dried grains with solubles (DDGS), copra meal (CM), palm kernel meal (PKM), and jatropha meal (JM).

Variable	DDGS		CM		PKM		JM		Tested feedstuffs (TF)				Physical forms (PF)		SEM	*P*-value		
	Mash	Pellet	Mash	Pellet	Mash	Pellet	Mash	Pellet	DDGS	CM	PKM	JM	Mash	Pellet		PF	TF	PF × TF
Dry matter	0.32^b^	0.38^a^	0.19^c^	0.19^c^	0.10^d^	0.15^c^	0.15^c^	0.20^c^	0.35^a^	0.19^b^	0.13^c^	0.18^b^	0.19^b^	0.23^a^	0.09	<0.001	<0.001	<0.001
Gross energy, MJ	0.37^b^	0.41^a^	0.23^e^	0.26^de^	0.19^f^	0.27^d^	0.33^c^	0.37^b^	0.39^a^	0.25^c^	0.23^c^	0.35^b^	0.28^b^	0.33^a^	0.07	<0.001	<0.001	<0.001
Nitrogen	−0.23^a^	−0.30^ab^	−0.36^b^	−0.32^b^	−1.03^e^	−0.53^c^	−1.15^f^	−0.92^d^	−0.27^a^	−0.34^b^	−0.78^c^	−1.03^d^	−0.69^b^	−0.52^a^	0.24	<0.001	<0.001	<0.001
Crude protein	−0.23^a^	−0.30^ab^	−0.36^b^	−0.32^b^	−1.03^e^	−0.53^c^	−1.15^f^	−0.92^d^	−0.27^a^	−0.34^b^	−0.78^c^	−1.03^d^	−0.69^b^	−0.52^a^	0.24	<0.001	<0.001	<0.001
Ether extract	0.81^d^	0.87^c^	0.20^h^	0.46^g^	0.52^f^	0.60^e^	0.97^a^	0.92^b^	0.84b	0.33^d^	0.56^c^	0.95^a^	0.62^b^	0.71^a^	0.12	<0.001	<0.001	<0.001
Crude ash	−2.27^c^	−0.97^a^	−3.05^d^	−1.33^b^	−4.15^e^	−1.38^b^	−4.81^f^	−4.03^e^	−1.62^a^	−2.19^b^	−2.76^c^	−4.42^d^	−3.57^b^	−1.93^a^	0.84	<0.001	<0.001	<0.001
Organic matter	0.40^ab^	0.39^b^	0.31^c^	0.25^d^	0.18^e^	0.21^e^	0.28^cd^	0.43^a^	0.40^a^	0.28^c^	0.20^d^	0.36^b^	0.29^b^	0.32^a^	0.08	<0.001	<0.001	<0.001
Total carbohydrate	0.66^b^	0.62^c^	0.51^e^	0.41^f^	0.39^g^	0.34^h^	0.59^d^	0.73^a^	0.64^b^	0.46^c^	0.36^d^	0.66^a^	0.54	0.52	0.08	0.181	<0.001	<0.001

*Note:* Means with different superscripts within a row of each classification (physical form or physical form × ingredient interaction) differ significantly (P < 0.05). SEM = standard

**Table 4:** Experiment 1: Effects of physical form (mash vs pellet) on metabolizable energy values of distillers dried grains with solubles (DDGS), copra meal (CM), palm kernel meal (PKM), and jatropha meal (JM).

Variable (MJ)	DDGS		CM		PKM		JM		Tested feedstuffs (TF)				Physical forms (PF)		SEM	*P*-value		
	Mash	Pellet	Mash	Pellet	Mash	Pellet	Mash	Pellet	DDGS	CM	PKM	JM	Mash	Pellet		PF	TF	PF × TF
AME	10.60^b^	11.44^a^	5.14^f^	6.34^e^	8.09^d^	8.43^d^	9.98^c^	11.06^ab^	11.02^a^	5.74^d^	8.26^c^	10.52^b^	8.45^b^	9.32^a^	2.05	<0.001	<0.001	<0.001
AMEn	10.60^b^	11.44^a^	5.14^f^	6.34^e^	8.09^d^	8.43^d^	9.98^c^	11.06^ab^	11.02^a^	5.74^d^	8.26^c^	10.52^b^	8.45^b^	9.32^a^	2.05	<0.001	<0.001	<0.001
TME	14.98^b^	15.72^a^	9.18^f^	10.27^e^	12.19^d^	12.30^d^	13.98^c^	15.03^b^	15.35^a^	9.72^d^	12.24^c^	14.50^b^	12.58^b^	13.33^a^	2.13	<0.001	<0.001	<0.001
TMEn	14.97^b^	15.71^a^	9.18^f^	10.27^e^	12.19^d^	12.30^d^	13.98^c^	15.03^b^	15.34^a^	9.72^d^	12.24^c^	14.50^b^	12.58^b^	13.33^a^	2.13	<0.001	<0.001	<0.001

*Note:* AME = apparent metabolizable energy; AMEn = nitrogen-corrected AME; TME = true metabolizable energy; TMEn = nitrogen-corrected TME. Means with different superscripts within a row of each classification (physical form or physical form × ingredient interaction) differ significantly (P < 0.05). SEM = standard error of the mean.

### Nutrient intake and output, digestibility, and nitrogen retention

As shown in Table 2, pelleting significantly increased dry matter (DM), gross energy (GE), ether extract (EE), organic matter (OM), and total carbohydrate intake compared with mash feeding (P < 0.05), while nitrogen and crude protein intake were not affected by physical form (P > 0.05). Among ingredients, DDGS generally exhibited higher nutrient intake values across most fractions, whereas PKM and CM showed comparatively lower intake, reflecting differences in nutrient density and physical characteristics. Nutrient output followed a similar pattern, with both feed ingredient and physical form significantly affecting excreta losses for most measured nutrients (P < 0.05). Overall, pelleting reduced nutrient losses, particularly for energy-related fractions; however, the response varied among ingredients, as indicated by significant ingredient × physical form interactions (P < 0.05). Dry matter digestibility (DMD) was significantly improved by pelleting across most ingredients (P < 0.05), with DDGS showing the highest digestibility and PKM the lowest. The significant interaction effect further indicated that the improvement in digestibility due to pelleting was strongly dependent on ingredient type. Apparent nitrogen retention (ANR) was negative across all treatments, reflecting net nitrogen losses inherent to the precision-fed assay. However, pelleting significantly improved ANR (less negative values) compared with mash feeding (P < 0.05). True nitrogen retention (TNR) showed a similar pattern, with higher values observed in pelleted diets and clear differences among ingredients (P < 0.05). DDGS consistently exhibited greater nitrogen retention than PKM and JM.

### Apparent total tract nutrient retention

Table 3 shows that pelleting significantly improved apparent total tract retention of dry matter, gross energy, ether extract, and organic matter across most ingredients (P < 0.05). In contrast, ash retention remained low or negative across treatments, indicating limited mineral utilization under the present assay conditions. Total carbohydrate retention was less responsive to processing and was more strongly influenced by ingredient type than by physical form. Significant ingredient × physical form interactions (P < 0.05) were observed for most retention variables, confirming that the effect of pelleting was not uniform across feedstuffs. Overall, DDGS showed the highest retention values, whereas PKM consistently exhibited the lowest.

### Metabolizable energy values

Metabolizable energy values were significantly affected by ingredient, physical form, and their interaction (Table 4). Pelleting consistently increased apparent metabolizable energy (AME), nitrogen-corrected AME (AMEn), true metabolizable energy (TME), and nitrogen-corrected TME (TMEn) compared with mash feeding (P < 0.05). Among ingredients, DDGS exhibited the highest metabolizable energy values, whereas CM showed the lowest across all energy metrics. PKM and JM showed intermediate responses, with JM generally higher than PKM. Significant interactions between ingredient and physical form indicated that the magnitude of improvement from pelleting varied among feedstuffs, with CM and JM showing the greatest response. AME and AMEn values were identical, as were TME and TMEn values, indicating minimal influence of nitrogen correction under the present experimental conditions.

### Experiment 2: Effects of β-mannanase supplementation on nutrient utilization and metabolizable energy

Tables 5–7 present the effects of β-mannanase supplementation, feed ingredient, and their interaction on nutrient intake, nutrient utilization, and metabolizable energy values of DDGS, copra meal (CM), palm kernel meal (PKM), and jatropha meal (JM). Overall, β-mannanase improved nutrient digestibility, reduced nutrient losses, and increased metabolizable energy, with responses varying markedly among ingredients, particularly between high-fiber (CM and PKM) and lower-fiber substrates.

**Table 5:** Experiment 2: Effects of β-mannanase supplementation on nutrient balance of distillers dried grains with solubles (DDGS), copra meal (CM), palm kernel meal (PKM), and jatropha meal (JM).

Variable	DDGS		CM		PKM		JM		Tested feedstuffs (TF)				Enzyme (E)		SEM	*P*-value		
	E (-)	E (+)	E (-)	E (+)	E (-)	E (+)	E (-)	E (+)	DDGS	CM	PKM	JM	-	+		E	TF	E×TF
Total nutrient intake (g)																		
Dry matter	25.19^e^	25.19^e^	27.29^b^	27.22^b^	26.75^c^	26.47^d^	27.50^a^	27.27^b^	25.19^d^	27.25^b^	26.61^c^	27.38^a^	26.68^a^	26.54^b^	0.40	0.006	<0.001	<0.001
Gross energy, MJ	0.57^f^	0.57^f^	0.60^e^	0.60^e^	0.64^c^	0.63^d^	0.72^a^	0.71^b^	0.57^d^	0.60^c^	0.64^b^	0.71^a^	0.63	0.63	0.02	0.356	<0.001	<0.001
Nitrogen	1.10^a^	1.10^a^	0.91^b^	0.90^c^	0.64^f^	0.63^g^	0.85^d^	0.84^e^	1.10^a^	0.91^b^	0.63^d^	0.81^c^	0.87	0.87	0.06	0.524	<0.001	<0.001
Crude protein	6.89^a^	6.89^a^	5.67^b^	5.65^c^	3.99^f^	3.94^g^	5.31^d^	5.27^e^	6.89^a^	5.66^b^	3.97^d^	5.29^c^	5.46	5.44	0.36	0.685	<0.001	<0.001
Ether extract	0.80^g^	1.68^d^	0.58^h^	1.03^f^	1.60^e^	1.93^c^	2.24^a^	2.13^b^	1.24^c^	0.81^d^	1.77^b^	2.18^a^	1.31^b^	1.69^a^	0.27	<0.001	<0.001	<0.001
Crude ash	1.69^e^	1.79^b^	1.44^f^	2.03^a^	0.99^h^	1.73^c^	1.00^g^	1.70^d^	1.74^a^	1.74^a^	1.36^b^	1.34^b^	1.28^b^	1.81^a^	0.18	<0.001	<0.001	<0.001
Organic matter	23.50^g^	23.40^h^	25.83^b^	25.19^e^	25.76^c^	24.74^f^	26.51^a^	25.58^d^	23.45^d^	25.52^b^	25.25^c^	26.04^a^	25.40^a^	24.73^b^	0.52	<0.001	<0.001	<0.001
Total carbohydrate	15.81^g^	14.83^h^	19.60^b^	18.51^e^	20.17^a^	18.86^d^	18.96^c^	18.18^f^	15.32^d^	19.06^b^	19.52^a^	18.57^c^	18.63^a^	17.60^b^	0.77	<0.001	<0.001	<0.001
Total nutrient output (g)																		
Dry matter	18.58^d^	15.13^e^	20.99^bc^	20.55^c^	23.39^a^	21.31^bc^	23.01^a^	21.66^b^	16.86^c^	20.77^b^	22.35^a^	22.33^a^	21.49^a^	19.66^b^	2.21	<0.001	<0.001	<0.001
Gross energy, MJ	0.34^e^	0.29^f^	0.40^cd^	0.39^d^	0.46^b^	0.42^c^	0.50^a^	0.46^b^	0.31^d^	0.40^c^	0.44^b^	0.48^a^	0.42^a^	0.39^b^	0.05	<0.001	<0.001	<0.001
Nitrogen	0.99^a^	0.65^d^	097^a^	0.84^c^	0.92^b^	0.83^c^	0.90^b^	0.85^c^	0.82^c^	0.91^a^	0.87^b^	0.87^b^	0.95^a^	0.79^b^	0.10	<0.001	<0.001	<0.001
Crude protein	6.21^a^	4.07^d^	6.06^a^	5.24^c^	5.74^b^	5.17^c^	5.62^b^	5.29^c^	5.14^c^	5.65^a^	5.46^b^	5.46^b^	5.91^a^	4.94^b^	0.60	<0.001	<0.001	<0.001
Ether extract	0.07^e^	0.05^f^	0.03^g^	0.02^h^	0.10^c^	0.09^d^	0.22^a^	0.17^b^	0.06^c^	0.02^d^	0.09^b^	0.20^a^	0.11^a^	0.08^b^	0.04	<0.001	<0.001	<0.001
Crude ash	0.20^c^	0.15^e^	0.20^c^	0.28^a^	0.16^d^	0.25^b^	0.16^d^	0.25^b^	0.17^c^	0.24^a^	0.21^b^	0.20^b^	0.18^b^	0.23^a^	0.04	<0.001	<0.001	<0.001
Organic matter	18.39^d^	14.98^e^	20.79^bc^	20.27^c^	23.23^a^	21.06^bc^	22.85^a^	21.41^b^	16.69^c^	20.53^b^	22.14^a^	22.13^a^	21.31^a^	19.43^b^	2.18	<0.001	<0.001	<0.001
Total carbohydrate	12.11^d^	10.87^e^	14.70^c^	15.02^c^	17.39^a^	15.80^b^	17.00^a^	15.95^b^	11.49^c^	14.86^b^	16.60a	16.47a	15.30^a^	14.41^b^	1.67	<0.001	<0.001	<0.001
DMD (%), Apparent nitrogen retained (ANR) and True nitrogen retained (TNR)																		
DMD	53.87^b^	67.60^a^	45.19^de^	50.10^c^	38.57^f^	42.77^de^	41.67^fe^	46.13^d^	60.74^a^	47.65^b^	40.67^d^	43.90^c^	44.82^b^	51.65^a^	8.83	<0.001	<0.001	<0.001
ANR	0.11^b^	0.45^a^	-0.06^e^	0.07^c^	-0.28^g^	-0.20^f^	-0.05^e^	-0.003^d^	0.28^a^	0.002^b^	-0.24^c^	-0.03^b^	-0.07^b^	0.08^a^	0.13	<0.001	<0.001	<0.001
TNR	1.04^b^	1.38^a^	0.86^e^	0.99^c^	0.65^g^	0.73^f^	0.88^e^	0.92^d^	1.21^a^	0.93^b^	0.69^d^	0.90^c^	0.86^b^	1.01^a^	0.13	<0.001	<0.001	<0.001

*Note:* DMD = dry matter digestibility; ANR = apparent nitrogen retention; TNR = true nitrogen retention. Means with different superscripts within a row of each classification (enzyme supplementation or enzyme × ingredient interaction) differ significantly (P < 0.05). SEM = standard error of the mean.

**Table 6:** Experiment 2: Effects of β-mannanase supplementation on apparent total tract retention of nutrients in distillers dried grains with solubles (DDGS), copra meal (CM), palm kernel meal (PKM), and jatropha meal (JM).

Variable	DDGS		CM		PKM		JM		Tested feedstuffs (TF)				Enzyme (E)		SEM	*P*-value		
	E (-)	E (+)	E (-)	E (+)	E (-)	E (+)	E (-)	E (+)	DDGS	CM	PKM	JM	-	+		E	TF	E×TF
Dry matter	0.54^b^	0.68^a^	0.49^cd^	0.50^c^	0.39^e^	0.46^d^	0.42^e^	0.46^d^	0.61^a^	0.49^b^	0.42^c^	0.44^c^	0.46^b^	0.52^a^	0.08	<0.001	<0.001	<0.001
Gross energy, MJ	0.41^b^	0.50^a^	0.34^c^	0.35^c^	0.29^d^	0.35^c^	0.31^d^	0.36^c^	0.45^a^	0.34^b^	0.32^c^	0.33^bc^	0.34^b^	0.39^a^	0.06	<0.001	<0.001	<0.001
Nitrogen	0.44^b^	0.68^a^	0.29^de^	0.39^c^	-0.01^g^	0.12^f^	0.26^e^	0.32^d^	0.56^a^	0.33^b^	0.05^d^	0.29^c^	0.24^b^	0.38^a^	0.14	<0.001	<0.001	<0.001
Crude protein	0.44^b^	0.68^a^	0.29^de^	0.39^c^	-0.01^g^	0.12^f^	0.26^e^	0.32^d^	0.56^a^	0.33^b^	0.05^d^	0.29^c^	0.24^b^	0.38^a^	0.14	<0.001	<0.001	<0.001
Ether extract	0.91^g^	0.97^b^	0.95^d^	0.98^a^	0.94^e^	0.96^c^	0.90^h^	0.92^f^	0.94^c^	0.97^a^	0.95^b^	0.91^d^	0.92^b^	0.96^a^	0.02	<0.001	<0.001	<0.001
Crude ash	0.88^b^	0.92^a^	0.86^cd^	0.86^c^	0.84^e^	0.86^cd^	0.84^e^	0.85^d^	0.90^a^	0.86^b^	0.85^c^	0.85^c^	0.86^b^	0.87^a^	0.02	<0.001	<0.001	<0.001
Organic matter	0.51^b^	0.66^a^	0.46^cd^	0.47^c^	0.37^f^	0.43^de^	0.40^e^	0.44^cd^	0.59^a^	0.47^b^	0.40^d^	0.42^c^	0.44^b^	0.50^a^	0.08	<0.001	<0.001	<0.001
Total carbohydrate	0.28^b^	0.46^a^	0.30^b^	0.28^b^	0.20^d^	0.26^bc^	0.17^d^	0.22^cd^	0.37^a^	0.29^b^	0.23^c^	0.19^d^	0.24^b^	0.30^a^	0.11	<0.001	<0.001	<0.001

*Note:* Means with different superscripts within a row of each classification (enzyme supplementation or enzyme × ingredient interaction) differ significantly (P < 0.05). SEM = standard error of the mean.

**Table 7:** Experiment 2: Effects of β-mannanase supplementation on metabolizable energy values of distillers dried grains with solubles (DDGS), copra meal (CM), palm kernel meal (PKM), and jatropha meal (JM).

Variable (MJ)	DDGS		CM		PKM		JM		Tested feedstuffs (TF)				Enzyme (E)		SEM	*P*-value		
	E (-)	E (+)	E (-)	E (+)	E (-)	E (+)	E (-)	E (+)	DDGS	CM	PKM	JM	-	+		E	TF	E×TF
AME	9.21^b^	11.32^a^	7.44^de^	7.71^cd^	6.98^e^	8.33^c^	8.03^cd^	9.29^b^	10.27^a^	7.58^c^	7.66^c^	8.66^b^	7.91^b^	9.16^a^	1.49	<0.001	<0.001	<0.001
AMEn	9.21^b^	11.32^a^	7.45^de^	7.71^cd^	6.98^e^	8.33^c^	8.03^cd^	9.29^b^	10.27^a^	7.58^c^	7.66^c^	8.66^b^	7.92^b^	9.16^a^	1.49	<0.001	<0.001	<0.001
TME	13.59^b^	15.70^a^	11.49^de^	11.77^d^	11.10^e^	12.51^c^	12.04^cd^	13.34^b^	14.65^a^	11.63^c^	11.80^c^	12.69^b^	12.06^b^	13.33^a^	1.51	<0.001	<0.001	<0.001
TMEn	13.59^b^	15.70^a^	11.49^de^	11.77^d^	11.10^e^	12.51^c^	12.04^cd^	13.34^b^	14.64^a^	11.63^c^	11.80^c^	12.69^b^	12.05^b^	13.33^a^	1.51	<0.001	<0.001	<0.001

*Note:* AME = apparent metabolizable energy; AMEn = nitrogen-corrected AME; TME = true metabolizable energy; TMEn = nitrogen-corrected TME. Means with different superscripts within a row of each classification (enzyme supplementation or enzyme × ingredient interaction) differ significantly (P < 0.05). SEM = standard error of the mean.

### Nutrient intake and output, digestibility, and nitrogen retention

As shown in Table 5, β-mannanase supplementation had no significant effect on dry matter, gross energy, nitrogen, or crude protein intake (P > 0.05), indicating that feed consumption remained comparable between treatments. However, ether extract intake and total carbohydrate intake were significantly affected by enzyme supplementation (P < 0.05), with responses differing among ingredients as reflected by significant enzyme × ingredient interactions. Nutrient output was significantly reduced by β-mannanase for most measured fractions, including dry matter, gross energy, nitrogen, crude protein, ether extract, organic matter, and total carbohydrates (P < 0.05). In contrast, ash output increased slightly with enzyme supplementation. Among ingredients, DDGS generally showed lower nutrient excretion, whereas PKM and CM exhibited higher losses, consistent with their higher fiber content and lower inherent digestibility. Dry matter digestibility (DMD) was significantly improved by β-mannanase across all ingredients (P < 0.05), with the greatest improvements observed in DDGS and CM. The significant enzyme × ingredient interaction indicated that the magnitude of response depended strongly on ingredient composition. Apparent nitrogen retention (ANR) and true nitrogen retention (TNR) were significantly increased by enzyme supplementation (P < 0.05). Although ANR remained negative in some cases, β-mannanase consistently reduced nitrogen losses (less negative values), indicating improved nitrogen utilization. TNR showed a similar pattern, with DDGS exhibiting the highest retention values and PKM the lowest.

### Apparent total tract nutrient retention

Table 6 shows that β-mannanase significantly improved apparent total tract retention of dry matter, gross energy, nitrogen, crude protein, ether extract, organic matter, and total carbohydrates (P < 0.05). The improvement was more pronounced in DDGS and CM, while PKM and JM showed moderate responses. Ash retention was also slightly increased by enzyme supplementation, although the effect was relatively small compared with organic nutrients. Significant enzyme × ingredient interactions were observed for all retention variables (P < 0.05), confirming that the response to β-mannanase was strongly dependent on feed ingredient characteristics.

### Metabolizable energy values

As presented in Table 7, β-mannanase supplementation significantly increased all metabolizable energy measures, including apparent metabolizable energy (AME), nitrogen-corrected AME (AMEn), true metabolizable energy (TME), and nitrogen-corrected TME (TMEn) (P < 0.05). The greatest increases were observed in DDGS and CM, whereas PKM and JM showed smaller but consistent improvements. Among ingredients, DDGS exhibited the highest metabolizable energy values across all metrics, while CM and PKM showed lower baseline energy availability. JM presented intermediate values but responded positively to enzyme supplementation. The similarity between AME and AMEn, as well as between TME and TMEn, indicates minimal influence of nitrogen correction under the present experimental conditions.

## Discussion

### Experiment 1: Effects of pelleting on nutrient utilization and metabolizable energy

The present study demonstrated that pelleting improved nutrient digestibility, nitrogen retention, and metabolizable energy of alternative poultry feed ingredients compared with mash forms. In particular, pelleting significantly enhanced dry matter digestibility, apparent nitrogen retention, true nitrogen retention, and true metabolizable energy (TME) across most ingredients evaluated. These findings are consistent with previous studies indicating that thermal and mechanical processing improves feed utilization by increasing substrate accessibility to endogenous enzymes, promoting starch gelatinization, and enhancing protein digestibility [13,14]. Structural disruption of feed matrices during pelleting likely reduces physical barriers to digestion, thereby facilitating more efficient nutrient hydrolysis and energy extraction.

The magnitude of response to pelleting varied among feed ingredients, reflecting differences in chemical composition and structural characteristics. Distillers dried grains with solubles (DDGS) and jatropha meal (JM) exhibited relatively greater improvements in metabolizable energy values, whereas responses in copra meal (CM) and palm kernel meal (PKM) were comparatively smaller. Such variation is likely associated with differences in fiber composition, nutrient density, and the extent to which nutrient fractions are physically encapsulated within the feed matrix.

CM and PKM contain substantial concentrations of β-mannans and other non-starch polysaccharides (NSPs), which are known to reduce nutrient availability through nutrient encapsulation and impaired digestive efficiency [4,15]. The negative apparent nitrogen retention values observed for some mash treatments further highlight the nutritional limitations associated with highly fibrous ingredients. Pelleting partially alleviated these constraints, likely through improvements in feed structure, particle cohesion, and nutrient accessibility, resulting in enhanced nutrient utilization and energy recovery [16].

Significant physical form × feed ingredient interactions observed for several response variables indicate that the effects of pelleting were ingredient-dependent. Therefore, the benefits of pelleting should not be assumed to be uniform across all alternative feed ingredients but rather interpreted in relation to the specific chemical and physical characteristics of each feedstuff.

### Experiment 2: Effects of β-mannanase supplementation on nutrient utilization and metabolizable energy

Supplementation with β-mannanase improved nutrient utilization and metabolizable energy values across the evaluated feed ingredients, with particularly pronounced responses observed in CM and PKM. These findings are consistent with the known mode of action of β-mannanase in degrading β-mannan-rich NSPs that limit nutrient digestion and absorption.

β-Mannans can increase intestinal viscosity, reduce interactions between digestive enzymes and nutrient substrates, and impair nutrient absorption [17,18]. Hydrolysis of β-mannans by exogenous β-mannanase reduces these anti-nutritional effects and promotes the release of encapsulated nutrients, thereby improving digestibility of dry matter, nitrogen, and energy. Previous studies have demonstrated that β-mannanase supplementation enhances growth performance, apparent metabolizable energy (AMEn), and nutrient retention in poultry, particularly when diets contain substantial quantities of NSP-rich ingredients [19,20].

A recent meta-analysis further confirmed that β-mannanase improves nutrient digestibility and energy utilization through reductions in digesta viscosity and improvements in intestinal morphology, including increased villus height and absorptive capacity [17]. Beyond viscosity reduction, β-mannanase may also influence endogenous digestive enzyme activity, microbial fermentation processes, and gut microbial populations, thereby contributing to improved nutrient absorption and digestive efficiency [18,21].

The present findings extend previous knowledge by demonstrating that β-mannanase supplementation improved not only apparent metabolizable energy values but also true metabolizable energy (TME) of individual feed ingredients. This observation confirms the effectiveness of β-mannanase in overcoming nutritional limitations associated with β-mannan-rich feed resources and supports its application in diets containing CM, PKM, and other fibrous ingredients.

Significant feed ingredient × enzyme interactions observed for several variables indicate that the magnitude of response to β-mannanase depended on ingredient composition. The larger improvements observed in CM and PKM are consistent with their higher β-mannan content and greater potential for enzymatic degradation of NSP fractions.

### Comparison between experiments

It should be noted that Experiments 1 and 2 were conducted independently to evaluate two distinct nutritional strategies, namely pelleting and β-mannanase supplementation. Consequently, direct numerical comparisons between experiments should be interpreted cautiously. Although similar feed ingredients and precision-fed rooster assay procedures were employed, differences in nutrient digestibility, nitrogen retention, and metabolizable energy values may reflect treatment-specific mechanisms, normal biological variation among birds across experimental rounds, and ingredient-dependent responses to processing. Pelleting primarily improves nutrient utilization through physical modification of feed structure, enhanced nutrient accessibility, and improved digestive efficiency [13,14], whereas β-mannanase acts through enzymatic hydrolysis of β-mannan-rich non-starch polysaccharides, thereby reducing anti-nutritional effects and improving nutrient availability [17,18]. Therefore, treatment effects are most appropriately interpreted within each experiment rather than through direct comparisons between experiments.

Because pelleting and β-mannanase supplementation were evaluated in separate experiments, the present study does not permit direct assessment of combined treatment effects or potential interactions between these nutritional strategies. Consequently, conclusions regarding additive or synergistic responses cannot be drawn from the current data. Future studies incorporating both pelleting and β-mannanase supplementation within a single factorial experimental design would be required to evaluate possible interactions between physical processing and enzymatic treatment. Similar recommendations have been proposed in feed evaluation research, where independent assessment of feed processing and enzyme supplementation does not allow formal determination of treatment interactions [16].

### Practical implications for alternative feed ingredients

Ingredient-specific responses observed in this study emphasize the importance of tailoring nutritional strategies according to feed composition. DDGS consistently exhibited relatively high digestibility and metabolizable energy values, in agreement with previous reports [22–24]. In contrast, CM and PKM showed more pronounced responses to β-mannanase supplementation, highlighting the importance of targeting NSP-degrading enzymes toward substrates rich in β-mannans [4].

Jatropha meal, despite its known anti-nutritional limitations, responded positively to both pelleting and β-mannanase supplementation when evaluated independently. These findings suggest that appropriate processing and nutritional interventions can substantially improve the feeding value of JM and may facilitate greater utilization of this alternative protein source in poultry nutrition [6].

## Conclusions

The results of the present study demonstrate that pelleting and β-mannanase supplementation independently improved nutrient digestibility, nitrogen utilization, and metabolizable energy of alternative poultry feed ingredients. Pelleting enhanced nutrient accessibility through physical modification of feed structure, whereas β-mannanase improved nutrient utilization through degradation of β-mannan-rich NSPs. The findings highlight the importance of selecting appropriate nutritional strategies according to ingredient characteristics and support the use of processing technologies and feed enzymes to improve the utilization efficiency of alternative feed resources in poultry production systems.

## Supporting information

S1 Data Raw individual bird data used for the calculation of nitrogen balance, metabolizable energy, and nutrient utilization in precision-fed Single Comb White Leghorn roosters. The dataset includes individual measurements of nitrogen intake, nitrogen excretion, apparent nitrogen retention (ANR), true nitrogen retention (TNR), feed intake, energy intake and output, apparent metabolizable energy (AME), nitrogen-corrected apparent metabolizable energy (AMEn), true metabolizable energy (TME), nitrogen-corrected true metabolizable energy (TMEn), and related variables for dried distillers grains with solubles (DDGS), palm kernel meal, copra meal, and detoxified jatropha meal provided in mash and pelleted forms. Each row represents one experimental bird used in the precision-fed rooster assay. (XLSX)

## References

[1] Keohavong B. Digital innovation integration into biotechnology for development of sustainable protein frontiers for poultry nutrition in a circular bioeconomy. Poult Sci. 2026;105(2):106276. doi: 10.1016/j.psj.2025.106276 41406819 PMC12950374

[2] Jin D, Tugiyanti E, Rimbawanto EA, Rosidi R, Widiyastuti T, Susanto A, et al. Effects of high-level dietary distillers dried grains with solubles supplemented with multienzymes on growth performance, nutrient utilization, intestinal morphology, and pellet quality in broiler chickens. Vet World. 2024;17(8):1943–54. doi: 10.14202/vetworld.2024.1943-1954 39328431 PMC11422655

[3] Shams-Eldin AM, Sayed-Ahmed A, Elbestawy AR, Elhamouly M, Saleh F, Fayed W, et al. Effects of dietary dried distillers’ grains with solubles and NSP enzyme supplementation on growth performance, intestinal morphology, immunity, and economic efficiency in broilers. Front Vet Sci. 2026;12:1752220. doi: 10.3389/fvets.2025.1752220 41669234 PMC12884538

[4] Punzalan JKM, Rosentrater KA. Copra meal: a review of its production, properties, and prospects. Animals (Basel). 2024;14(11):1689. doi: 10.3390/ani14111689 38891735 PMC11171203

[5] Singh AK, Kim WK. Effects of dietary fiber on nutrients utilization and gut health of poultry: a review of challenges and opportunities. Animals (Basel). 2021;11(1):181. doi: 10.3390/ani11010181 33466662 PMC7828824

[6] Oskoueian E, Oskoueian A, Shakeri M, Jahromi MF. Benefits and challenges of jatropha meal as novel biofeed for animal production. Vet Sci. 2021;8(9):179. doi: 10.3390/vetsci8090179 34564573 PMC8472097

[7] Jha R, Mishra P. Dietary fiber in poultry nutrition and their effects on nutrient utilization, performance, gut health, and on the environment: a review. J Anim Sci Biotechnol. 2021;12(1):51. doi: 10.1186/s40104-021-00576-0 33866972 PMC8054369

[8] Nusairat B, Odetallah N, Tsai C-Y, Wang J-J. Effect of dietary β-mannanase supplementation on broiler performance. Poult Sci. 2024;103(3):103452. doi: 10.1016/j.psj.2024.103452 38262336 PMC10835434

[9] Svihus B, Uhlen AK, Harstad OM. Effect of starch granule structure, associated components and processing on nutritive value of cereal starch: a review. Animal Feed Sci Tech. 2005;122(3–4):303–20. doi: 10.1016/j.anifeedsci.2005.02.025

[10] Sibbald IR. The TME system of feed evaluation: Methodology, feed composition data, and bibliography. Animal Research Centre Technical Bulletin. 1986; 1: 1–74.

[11] Dudley-Cash WA. A landmark contribution to poultry science--a bioassay for true metabolizable energy in feedingstuffs. Poult Sci. 2009;88(4):832–4. doi: 10.3382/ps.2008-00506 19276429

[12] AOAC International. Official methods of analysis. 18th ed. Gaithersburg (MD): AOAC International; 2005.

[13] Svihus B. The gizzard: function, influence of diet structure and effects on nutrient availability. Worlds Poult Sci J. 2011; 67(2): 207–24. doi: 10.1017/S0043933911000249

[14] Hossain ME. Enhanced bioavailability of nutrients in pelleted feeds: Implications for performance, health, and meat quality of the broiler chicken. Res Rev J Health Prof. 2025; 15(01): 12–32.

[15] Ong WL, Chan KL, Suwanto A, Li Z, Ng K-H, Zhou K. Hydrolysis of palm kernel meal fibre using a newly isolated Bacillus subtilis F6 with high mannanase activity. Bioresour Bioprocess. 2024;11(1):113. doi: 10.1186/s40643-024-00826-9 39720965 PMC11669640

[16] Adeola O, Cowieson AJ. Board-invited review: opportunities and challenges in using exogenous enzymes to improve nonruminant animal production. J Anim Sci. 2011;89(10):3189–218. doi: 10.2527/jas.2010-3715 21512114

[17] Kim HW, Kwon CH, Lee JH, Kang MS, Kil DY. Effect of dietary β-mannanase supplementation on growth performance, intestinal morphology, digesta viscosity, and nutrient utilization in broiler chickens: Meta-analysis and meta-regression. Anim Biosci. 2024;37(12):2113–25. doi: 10.5713/ab.24.0459 39210792 PMC11541010

[18] Zhang L, Huan H, Zhang K, Tu Y, Yan J, Zhang H, et al. Effects of β-mannanase supplementation on growth performance, digestive enzyme activity, cecal microbiota, and short-chain fatty acid production in broilers. J Anim Sci. 2024. doi: 10.1093/jas/skae184 PMC11345513 38980728

[19] Sacakli P, Ramay MS, Ahsan U, Gebes ES, Harijaona JA, Fickler A, et al. Effect of dietary β-mannanase supplementation on growth performance and nutrient retention in broiler chickens fed corn-soybean meal-based diets with low energy and amino acid density. Poult Sci. 2024;103(12):104475. doi: 10.1016/j.psj.2024.104475 39510008 PMC11577199

[20] Yu M, Oketch EO, Nawarathne SR, Chathuranga NC, Maniraguha V, Sta Cruz BG, et al. Metabolizable energy and amino acid-deficient diets supplemented with β-mannanase in response to growth performance, intestinal health, and immune response in broilers. Poult Sci. 2025;104(7):105222. doi: 10.1016/j.psj.2025.105222 40318546 PMC12123341

[21] Balasubramanian B, Ingale SL, Park JH, Rathi PC, Shanmugam S, Kim IH. Inclusion of dietary β-mannanase improves performance and ileal digestibility and reduces ileal digesta viscosity of broilers fed corn-soybean meal based diet. Poult Sci. 2018;97(9):3097–101. doi: 10.3382/ps/pey157 29771358

[22] Caldas JV, Hilton K, Mullenix G, Du X, England JA, Coon CN. Corn distillers dried grains with solubles: nutrient analysis, metabolizable energy, and amino acid digestibility in broilers. J Appl Poult Res. 2020; 29(4): 1068–83. doi: 10.1016/j.japr.2020.09.015

[23] Goo D, Lee DJ, Kim Y, Kim WK. The effects of dietary levels of corn distillers dried grains with solubles and supplementation of valine and isoleucine on growth performance, intestinal health, and cecal microbiome in Ross 708. Poult Sci. 2025;104(12):105910. doi: 10.1016/j.psj.2025.105910 41037885 PMC12523081

[24] Walker H, Vartiainen S, Apajalahti J, Taylor-Pickard J, Nikodinoska I, Moran CA. The effect of including a mixed-enzyme product in broiler diets on performance, metabolizable energy, phosphorus and calcium retention. Animals (Basel). 2024;14(2):328. doi: 10.3390/ani14020328 38275788 PMC10812510

